# A Tunable Glycosaminoglycan–Peptide Nanoparticle Platform for the Protection of Therapeutic Peptides

**DOI:** 10.3390/pharmaceutics16020173

**Published:** 2024-01-25

**Authors:** Harkanwalpreet Sodhi, Alyssa Panitch

**Affiliations:** 1 Department of Biomedical Engineering, University of California, Davis, CA 95616, USA; hssodhi@ucdavis.edu; 2 Wallace H. Coulter Department of Biomedical Engineering, Georgia Institute of Technology and Emory University, Atlanta, GA 30332, USA

**Keywords:** nanoparticle, glycosaminoglycan, peptide, inflammation, tunable

## Abstract

The popularity of Glycosaminoglycans (GAGs) in drug delivery systems has grown as their innate ability to sequester and release charged molecules makes them adept in the controlled release of therapeutics. However, peptide therapeutics have been relegated to synthetic, polymeric systems, despite their high specificity and efficacy as therapeutics because they are rapidly degraded in vivo when not encapsulated. We present a GAG-based nanoparticle system for the easy encapsulation of cationic peptides, which offers control over particle diameter, peptide release behavior, and swelling behavior, as well as protection from proteolytic degradation, using a singular, organic polymer and no covalent linkages. These nanoparticles can encapsulate cargo with a particle diameter range spanning 130–220 nm and can be tuned to release cargo over a pH range of 4.5 to neutral through the modulation of the degree of sulfation and the molecular weight of the GAG. This particle system also confers better in vitro performance than the unencapsulated peptide via protection from enzymatic degradation. This method provides a facile way to protect therapeutic peptides via the inclusion of the presented binding sequence and can likely be expanded to larger, more diverse cargo as well, abrogating the complexity of previously demonstrated systems while offering broader tunability.

## 1. Introduction

Glycosaminoglycans (GAGs) consist of a family of long, unbranched polysaccharide chains composed of the chondroitin sulfates—including dermatan sulfate (DS) -, heparin sulfate, keratin sulfate, and hyaluronic acid. GAGs are composed of repeating disaccharide units and are found throughout the tissue extracellular matrix, and often found in higher concentrations in the glycocalyx. The GAG disaccharide subunits contain one hexuronic acid and one amino sugar linked by glycosidic bonds, with some units decorated with negatively charged sulfate groups [1]. The sulfate groups confer a charge that allows GAGs to sequester growth factors and other signaling molecules. The ability of GAGs to interact with membrane receptors, ECM proteins, chemokines, cytokines, and enzymes is crucial for the maintenance of homeostasis but, conversely, also implicates them in the progression of many diseases [2,3,4,5].

These GAG-protein interactions have made them appealing for use in therapeutic development and delivery. They can be incorporated into hydrogel and particle systems to electrostatically sequester and release charged molecules, including growth factors and small molecule therapeutics, in a controlled manner, similar to their function in vivo [6,7]. Heparin-based hydrogels have been employed increasingly as growth factor carriers and scaffolds for tissue regeneration due to their affinity for growth factors and, more broadly, their ability to couple with charged molecules [8,9,10,11]. More recently, interest has grown in the development of GAG-functionalized and GAG-containing nanoparticles for incorporation into hydrogels or for systemic delivery [10,11,12,13,14,15,16,17]. Heparin has been used to form particles loaded with paclitaxel, a common cancer therapeutic, via covalent binding using a cleavable amino acid linker to support incorporation and release [17]. Nanoparticles formulated with chondroitin sulfate and chitosan, a cationic polysaccharide derived from the chitin of shellfish [18], have been loaded with platelet lysate. The lysate is a solution rich in growth factors, proteins, cytokines, and chemokines that is administered to treat a myriad of different diseases [14]. Electrolyte complexes of hyaluronic acid and heparin functionalized with chitosan have also been shown to form nanoparticles, which can be loaded with growth factors such as Fibroblast Growth Factor II as well as Bone Morphogenetic Protein 2 [11,15].

These systems have some limitations, however, both due to their complexity and reliance on multiple synthetic and organic polymers, covalent linkages, and chemical modification to the polymers and, in some cases, to the cargo as well. The use of synthetic polymers introduces the potential for byproducts and unintended toxicities, which complicate their use in vitro [19]. Using multiple polymers or chemically modifying particle constituents increases production time and complexity and, therefore, cost. Applications of these methods to the encapsulation of peptides are lacking, despite peptides being highly specific and effective therapeutics [20,21] whose main drawback is that they are rapidly degraded in vivo (and in vitro) by constitutively expressed proteases and peptidases when not encapsulated [22]. Peptide delivery is generally relegated to complex, synthetic nanoparticle systems, which slows their development and path to market [19,23,24,25,26,27].

We have previously demonstrated a GAG-peptide nanoparticle system which circumvents these issues of complexity, while still augmenting the efficacy of the encapsulated peptide cargo [2]. This was achieved by vortexing or electrospraying dermatan sulfate with a cationic peptide containing both a cell-penetrating amino acid sequence derived from the heparin-binding domain of Anti-Thrombin III (KAFAK) and an anti-inflammatory MK2 inhibitor sequence (MK2i). This system is facile and takes advantage of the opposing charges on DS and KAFAK-MK2i to form nanoparticles. These particles were shown to have a long shelf life and be stable against sonication and highly acidic pH. They also improved the efficacy of KAFAK-MK2i anti-inflammatory peptide, likely because they conferred protection against enzymes present in fetal calf serum and in the body [2].

Here, we present an investigation into the interaction of KAFAK with GAGs as a function of charge ratio and GAG molecular weight to obtain a deeper understanding of this assembly behavior; we also used another cationic cell-penetrating peptide to determine whether the assembly method can be used generally for cationic species. Further, we attached KAFAK to different cargo to determine whether KAFAK can be used to form particles with GAGs to deliver other cargo. Overall, we present a nanoparticle platform which allows for facile assembly based on electrostatic interactions and control over particle size and release behavior.

**Figure 1:**
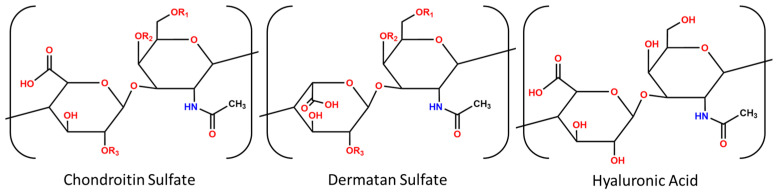
Repeating disaccharide units of dermatan sulfate, hyaluronic acid, and chondroitin sulfate, which are similar to hyaluronic acid but contain sulfates. Chondroitin sulfate and dermatan sulfate differ in the conformation of one carboxylic acid moiety (equatorial vs. axial configuration). “R” indicates a potential sulfation point.

To do this, hyaluronic acid (HA), an unsulfated GAG, served as a “blank slate” GAG with no anionic sulfate groups [28], to which we methodically added sulfates using a one-step reaction to control the charge ratio between sulfated HA and KAFAK. We also modulated the molecular weight (MW) of the HA polymer. HA was used because, although it lacks the anionic sulfates found in the chondroitin sulfate family of GAGs, it is structurally analogous to DS, with the only difference being that HA has one carboxylic acid moiety in an equatorial position, as opposed to the axial position found in DS (see Figure 1) [28]. We then investigated how GAG MW and the degree of sulfation affected particle diameter, zeta potential, minimum stable pH, and biological efficacy. We also tested complexation with a different MK2 inhibitor peptide, termed YARA, that is also cationic but not known to be highly heparin-binding. We found that charge ratio and MW can be used to tune the control of particle formation, disassembly, and size with the KAFAK peptide, but not the YARA peptide. Lastly, we showed that these nanoparticles conferred better in vitro performance than the unencapsulated peptide. To show this, we used a trypsin-based model of accelerated in vitro degradation to demonstrate that these particles protected their cargo from enzymatic degradation, and this likely serves as the reason for their improved efficacy in vitro. This glycosaminoglycan-based particle system has the potential to encapsulate and protect a myriad of cargo while remaining facile and cheap, and abrogating the need for synthetic and polymeric solutions which complicate pharmacokinetics. While some such electrostatic, two-component, nanoparticle systems exist [25], none exist that utilize an organic polymer.

## 2. Materials and Methods

### 2.1. Peptide Synthesis

The MK2i peptide (sequence KAFAKLAARLYRKALARQLGVAA) was synthesized using a CEM Liberty Blue (CEM Corporation, Matthews, NC, USA) automated peptide synthesizer with microwave acceleration using solid-state peptide synthesis on medium-loading rink amide resin. The amino acids were linked using N-hydroxybenzotriazole (HoBt)/N, N′-diisopropylcarbodiimide (DIC) chemistry for the first reaction and 2-(1Hbenzotriazole-1-yl)-1,1,3,3-tetramethyluronium hexafluorophosphate (HBTU), lutidine chemistry for the second step (Sigma-Aldrich, Saint Louis, MO, USA). Amino acids with an FMOC protective group (AAPPTec, Louisville, KY, USA) were dissolved in synthesis-grade dimethylformamide (DMF) (Fisher Chemical, Hampton, NH, USA) at a final concentration of 0.2 M. Synthesis was carried out at 50 °C using microwave heating for 60 min, with coupling steps repeated twice for each arginine addition in the sequence. For the testing of fluorescein-labeled peptide, fluorescein isothiocyanate (Sigma-Aldrich, Saint Louis, MO, USA) was conjugated to the N-terminus of the KAFAK-MK2i peptide during automated peptide synthesis. The peptide was then cleaved away from the rink amide resin using a cocktail consisting of 4.4 mL of trifluoroacetic acid, 0.25 of mL phenol, 0.10 mL of triisopropylsilane (Fisher Scientific, Hampton, NH, USA/Acros Organics, Antwerp, Antwerp Province, Belgium), and 0.25 mL of MilliQ water. The resin was soaked and agitated in cleavage cocktail for 3 h on a tube rotator (Labnet international, Edison, NJ, USA). The fully cleaved and deprotected peptide was then precipitated from solution using 20 mL of 4 °C diethyl ether (Fisher Chemical, Hampton, NH, USA). Peptide was isolated from solution by centrifugation at 4000 rpm for 2 min. The diethyl ether was decanted, and the peptide was re-suspended and washed three times consecutively. Following this, the crude peptide was allowed to dry under vacuum for a minimum of 18 h. The dried, crude peptide was then dissolved in MilliQ water and purified using reverse-phase chromatography (ÄKTA Explorer, GE Healthcare, Chicago, IL, USA). Acetonitrile (Fisher Chemical, Hampton, NH, USA) and ultrapure water with 0.1% trifluoroacetic acid were used for purification, along with a C18 prep-scale reverse phase column (Phenomenex, Torrance, CA, USA). Finally, the molecular weight and purity of the peptide were assessed using a 4800 Plus MALDI TOF/TOF™ Analyzer (Applied Biosystems, Waltham, MA, USA). The final product was then frozen at −80 °C and lyophilized on an SP Scientific (Warminster, PA, USA) FreezeMobile lyophlizer until completely dry.

### 2.2. Sulfation of Hyaluronic Acid

Hyaluronic acid of 10 kDa, 20 kDa, 60 kDa, 100 kDa, and 200 kDa (Lifecore Biomedical, Menlo Park, CA, USA) molecular weights were each sulfated by dissolving in formamide at a starting concentration of 10 mg/mL in a glass, 20 mL scintillation vial with stir bar. The vial was heated in an oil bath maintained at 60 °C using a hot plate equipped with a probe (IKA Works, Staufen, Baden-Württemberg, Germany). HA was allowed to dissolve with vigorous stirring for approximately 15 min at 60 °C until the solution was completely clear. The solution was then aliquoted into more vials for various different degrees of sulfation and diluted to a final concentration of 2 mg/mL. Chlorosulfonic acid (Sigma-Aldrich, Saint Louis, MO, USA) was then added to each solution dropwise at a volume ranging from 10 to 100 µL depending on degree of sulfation. Target degrees of sulfation were 60, 80, 100, 120, and 140 percent relative to DS (1.1 sulfate group per saccharide unit). The reaction was allowed to continue at 60 °C for two hours, at which point 4 °C acetone (Sigma-Aldrich, Saint Louis, MO, USA) was added to stop the reaction and precipitate the sulfated HA. Sulfated HA was then washed three times in a 50 mL conical tube using cold acetone and centrifuged at 4000 rpm for 5 min in an Eppendorf 5810R centrifuge (Eppendorf, Hamburg, Germany) and decanted. The pellet of sulfated HA was dried overnight and dissolved in ultrapure water using sonication for 15 min, at which point the total volume was brought up to 40 mL and tangential flow filtration was used to purify at a flow rate of 100 mL/min and backpressure of 18 psi with a 10 kDa molecular weight cutoff column on a Spectrum Labs KrosFlo KR2i (Spectrum Labs, Rancho Dominguez, CA, USA). It is important to note that each stock of HA has a size range, with 10 kDa HA ranging from 10 to 20 kDa, 20 kDa ranging from 21 to 40 kDa, 60 kDa ranging from 66 to 99 kDa, 100 kDa ranging from 100 to 175 kDa, and 200 ranging from 151 to 300 kDa.

### 2.3. Measurement of Degree of Sulfation

Sulfated HA was dissolved in 1 M hydrochloric Acid (Sigma-Aldrich, Saint Louis, MO, USA) at a concentration of 1 mg/mL in a 1.5 mL Eppendorf tube and heated on a heating block for three hours at 100 °C. Then, 20 µL of each sample was mixed with 380 µL of trichloroacetic acid at a concentration of 4% and 100 µL of barium chloride + gelatin solution (Sigma-Aldrich, Saint Louis, MO, USA). BaCl gelatin solution was prepared by dissolving gelatin in water at 70 °C with vigorous stirring at a concentration of 0.5% *w*/*v*. The gelatin solution was allowed to sit overnight, after which BaCl was dissolved at a concentration of 0.5% *w*/*v* with vigorous stirring and no heating. The solution was allowed to sit for at least 2–3 h before use and discarded and remade after 7 days. The mixture of sample and BaCl + gelatin was allowed to sit for 20 min, after which it was vortexed again and 200 µL of each sample was pipetted into a black, 96-well, clear bottom, non-binding plate and the absorbance was measured at 360 nm on a SpectraMax M5 plate reader (Molecular Devices, San Jose, CA, USA).

### 2.4. Particle Synthesis

**Figure 2:**
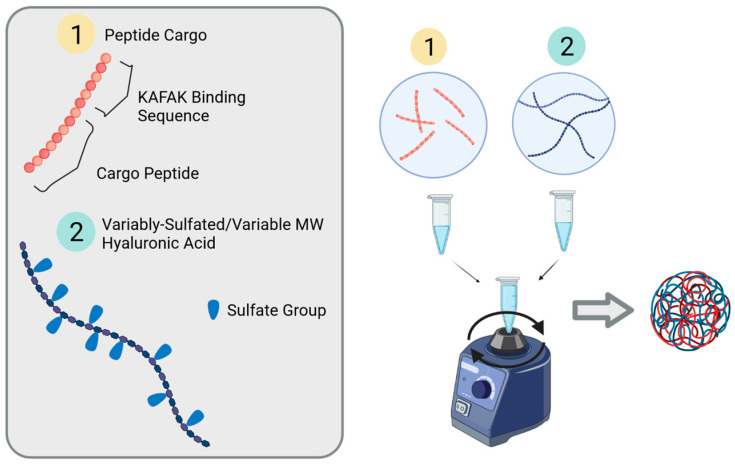
General principle of sulfated HA + KAFAK-MK2i nanoparticle synthesis and formulation.

Sulfated HA and KAFAK and YARA were dissolved in ultrapure water, each at a concentration of 1 mg/mL, and sonicated in an Elmasonic P30H bath sonicator at 100 watts for 15 min. After dissolving, 500 µL of SHA solution was added to a new tube, 500 µL of peptide solution was added, and the vial was vortexed vigorously for ten seconds (see Figure 2). This mixture was then allowed to sit at 4 °C for 24 h. The solution was then analyzed using dynamic light scattering as described in the following section, and 10X phosphate-buffered saline was then added for a final concentration of 1X and reanalyzed.

### 2.5. Dynamic Light Scattering Measurements of Size and Zeta Potential

Particle size was measured using dynamic light scattering on a Malvern ZetaSizer with 633 nm laser and a DTS0012 polystyrene cuvette (Malvern P analytical, Malvern, Worcestershire, UK) with a pathlength of 1 cm. Viscosity, material absorption, and refractive index were set to 1.330, 0.010, and 0.8872 cP, respectively, using Mei theory and the general purpose, non-negative least squares regression for calculation of the z-average diameter. All samples were equilibrated to 21 °C for 2 min and all readings were taken three times per sample. Only samples with a unimodal size distribution and a PDI below 0.2 were accepted for further analysis.

### 2.6. Particle Size Dynamics at Varying pH

Particles with varying molecular weight HA and varying degrees of sulfation were synthesized as previously described. The initial pH of the solution was measured. The pH was adjusted using 1 M hydrochloric acid (Sigma-Aldrich, Saint Louis, MO, USA) in pH increments of 0.5 and the size distribution and zeta potential were measured at each increment as previously described. Samples which went below the pH target during this process were discarded and the process was started over.

### 2.7. Enzymatic Degradation Studies

Dermatan sulfate (Celsus Laboratories, Cincinnati, OH, USA)+ KAFAK-MK2i particles were synthesized as previously described. TrypLE Express (Thermo Fisher Scientific, Waltham, MA, USA) dissociation reagent solution (Recombinant Trypsin substitute + EDTA) was added to samples of KAFAK-MK2i only and DS-KAFAK-MK2i nanoparticles in ultrapure water for a final concentration of 10%. Samples were spun on an orbital plate shaker (IKA Works, Staufen, Baden-Württemberg, Germany) at 180 rpm for ten minutes at 37 °C, at which point a sample was taken and plated for MALDI-TOF analysis to qualitatively measure the presence of complete peptide and peptide fragments. Briefly, one microliter of a 4 mg/mL solution α-cyano-4-hydroxycinnamic acid (dissolved in 59.95% acetonitrile, 39.95% water, and 0.1% TFA) (Sigma Aldrich, Saint Louis, MO, USA) was spotted onto the MALDI plate and 1 µL of sample was added to this spot, while repeatedly pipetting up and down to thoroughly mix the sample with the matrix.

### 2.8. In Vitro Assessment of Particle Efficacy

Immortalized human keratinocytes (AddexBio Technologies, San Diego, CA, USA) were cultured in a T75 tissue culture flask (Sarstedt AG & Co, Nümbrecht, North Rhine-Westphalia, Germany) using DMEM supplemented with 2% fetal bovine serum (Promocell GmbH, Heidelberg, Baden-Wurttemberg, Germany) from a starting density of 500,000 cells/mL. Cells were then lifted from the flask using TrypLE dissociation reagent (Thermo Fisher Scientific, Waltham, MA, USA) and centrifuged to a pellet. The TrypLE was aspirated, and the pellet was resuspended in DMEM with fetal calf serum. Cells were pelleted once more and DMEM was aspirated to remove any remaining TrypLE. Cells were resuspended in DMEM + serum and seeded on a Corning 96-well tissue culture plate at 10,000 cells per well in a volume of 200 µL of DMEM + serum per well.

Upon initiation of experiments, unstimulated control cells were given 200 µL of fresh media. Artificially inflamed treatment groups were given 200 µL per well of DMEM + serum containing 2.5 ng/mL of IL-1β and 0.3 ng/mL of TGF-β (Peptrotech Inc., Cranbury, NJ, USA). Inflamed cells were treated with this cytokine cocktail for 1 h, after which point the cells were washed with Hank’s Buffered Salt solution (Sigma-Aldrich, Saint Louis, MO, USA) twice to remove added cytokine and provide fresh DMEM+serum media.

Inflamed and uninflamed keratinocytes were treated with PBS (negative control), KAFAK-MK2i or successful SHA-peptide particle candidates, all with a final concentration of 1 mg/mL of KAFAK-MK2i. All experimental therapeutics were filtered with a 13 mm, 0.2 µM nylon filter (Pall Corporation, New York City, NY, USA). Cells were grown and incubated during experiments at 37 °C at 5% CO_2_ in a cell incubator. Media was sampled at 6 h and 24 h to evaluate drug performance as a function of time. The amount of inflammatory cytokine IL-6 secreted by the keratinocytes during incubation was measured using the Meso Scale Diagnostics V-plex 4-panel human inflammatory cytokine electrochemiluminescent assay (Meso Scale Diagnostics, Rockville, MD, USA).

## 3. Results and Discussion

### 3.1. The KAFAK Binding Sequence and Cargo Loading

**Figure 3:**
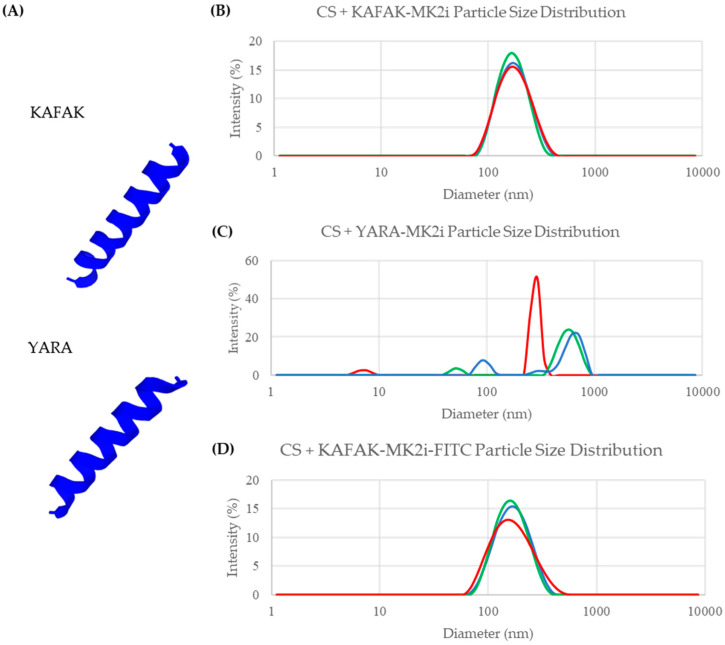
(**A**) YARA and KAFAK peptide sequences both generate an alpha helix, as shown in these computational models of KAFAK and YARA peptide structures. (**B**) When using CS, a GAG very similar to DS and identical to sulfated HA, to formulate nanoparticles, instead of the DS used previously, KAFAK-MK2i forms particles as shown by unimodal and narrowly distributed size distribution generated using dynamic light scattering (red, green, and blue represent replicate measurements of the same sample). (**C**) When combined with CS, the YARA-MK2i peptide does not form stable particles, as evidenced by the transient, multimodal size distribution. (**D**) When KAFAK-MK2i is conjugated with fluorescein (FITC), it forms nanoparticles of similar size and quality to KAFAK-MK2i alone, indicating that a variety of cargo with differing charges can be loaded into nanoparticles using the KAFAK binding sequence.

We hypothesized that the KAFAK amino acid sequence conferred the peptide with the ability to assemble with GAGs into nanoparticles, given that KAFAK is derived from the heparin binding domain of anti-thrombin III and has inherent GAG-binding ability. To test this, we investigated the ability of a similar peptide sequence, termed YARA, to form particles when attached to the same MK2 inhibitor peptide sequence as cargo. YARA is a cell-penetrating peptide sequence derived from HIV TAT [29], but not known to be strongly GAG-binding. The KAFAK and YARA binding sequences and their cargo are shown below in Table 1. Because we had previously shown that KAFAK formed particles with members of the chondroitin sulfate family of GAGs, including DS and chondroitin-6 sulfate (CS-6), we tried to form particles using YARA and CS-6. When mixed with CS-6, KAFAK-MK2i formed particles (Figure 3B), whereas YARA-MK2i did not form stable particles (Figure 3C). This was despite KAFAK and YARA’s similar number of charged amino acids (Table 1) and indicated that the KAFAK sequence was responsible for particle formation, not general charge interactions or the MK2i sequence.

To determine if this difference in binding was due to conformational differences between the peptides, computational modeling of the tertiary structure of KAFAK and YARA peptides was carried out using the PEP-FOLD3 de novo peptide structure prediction system (developed and provided free of charge by Paris Diderot University in Paris, France) [30,31]. Simulations showed that both peptide sequences likely adopt an alpha-helical structure (Figure 3A), suggesting tertiary structure alone is not the variable at play. This is supported by previous studies using circular dichroism to assess the structure of YARA and KAFAK [32]. The answer may lie, therefore, in the exact number, identity, and spatial orientation of the cationic charges in each peptide. KAFAK has six cationic amino acid residues, compared to five in YARA. Further, α-helixes have 3.6 amino acid residues per turn [33,34], and thus the distance between cationic residues within the polypeptide chain may result in a preferred facial charge alignment for GAG binding in KAFAK vs. YARA sequences.

These findings align with expectations based on the origins of the KAFAK sequence, which is directly derived from the heparin-binding domain of antithrombin III [35], a protein which is evolutionarily optimized to bind to heparin and, through structural similarity, other sulfated GAGs. By covalently linking the KAFAK sequence to cargo of similar mass to the MK2i peptide, one can likely form electrostatic particles with most sulfated GAGs. To test this hypothesis, we attempted to make particles using KAFAK linked to MK2i tagged with fluorescein, to represent a different cargo, and to ascertain if changes to the cargo peptide would abrogate particle formation. Fluorescein, which increased the peptide MW by 13.4%, is an anionic molecule that has been shown to affect protein binding with the glycocalyx [36]. Therefore, it represents a cargo that should inhibit particle formation if particle formation is very sensitive to the cargo of choice. As shown in Figure 3D, fluorescein-labelled KAFAK-MK2i forms particles with CS-6 just as well as MK2i alone, with unimodally sized particles and a low PDI, making this nanoparticle platform more broadly applicable, supporting KAFAK-MK2i delivery and the delivery of other cargo linked to KAFAK, or potentially other heparin-binding peptides.

### 3.2. Charge Ratio, Molecular Weight, and Tunability

Having established that KAFAK was required for particle formation with GAGs and that KAFAK supported particle formation with different cargo, we investigated whether we could tune particle size and stability. Tunability would allow the modulation of particle size and the pH at which the particles release their cargo to tailor particle properties to the application needs. We turned to the charge ratio between the two molecules to examine this. In order to modulate the charge ratio between KAFAK-MK2i and the GAG, HA of 10 kilodaltons (kDa), 20 kDa, 60 kDa, 100 kDa, and 200 kDa molecular weight (MW), were chosen to represent a spread of molecular weights close to, above, and below the molecular weight of both the native dermatan sulfate and chondroitin sulfate used in previous studies (approximately 40 kDa) [2]. We targeted and synthesized sulfated HA (SHA) with 60, 80, 100, 120, and 140% of the average sulfate content of DS for each molecular weight of HA investigated. This allowed us to modulate particle formation over a broad range of polymer MW and degrees of sulfation and to use our findings with DS as a benchmark [2]. SHA was then sonicated in solution and combined with KAFAK, as previously described [28]. Particle formation was assessed using YARA-MK2i and KAFAK-MK2i. YARA-MK2i was included to see if altering the sulfate charge density on the GAG would support particle formation generally with cationic peptides.

**Figure 4:**
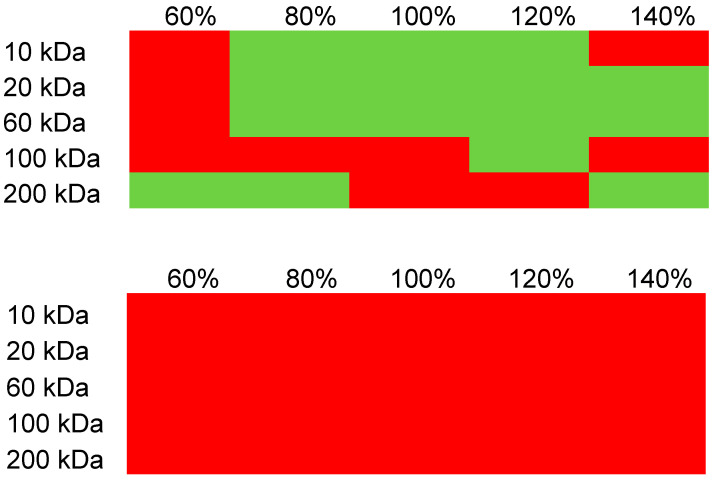
Matrix indicating particle stability. Red—low to no stability. Green—stable. Top KAFAK-MK2i, bottom YARA-MK2i.

Nanoparticle formulations were assessed for size using dynamic light scattering and those that exhibited a unimodal size distribution and a PDI of 0.2 or less were retained for additional studies. As shown in Figure 4, there was a wide range of MWs and degree of sulfation of the SHA formed particles with KAFAK-MK2i. Groups shown in red in the matrix of Figure 4 formed particles that aggregated during overnight equilibration or aggregated upon the addition of PBS and were not studied further. Groups shown in green in Figure 4 remained stable. However, when the KAFAK sequence is substituted for YARA, no groups successfully formed particles which met our suitability and stability requirements.

These data confirmed that cationic charge alone for the cargo peptide was insufficient to support particle formation. It also demonstrated that both GAG MW and degree of sulfation contributed to particle stability, with a lower degree of sulfation failing to support particle stability, except at the higher SHA molecular weight. In addition, as SHA MW increased, particle stability generally decreased with the higher degrees of sulfation, with the exception of the 200 kDa and 140% sulfation group and the 100 kDa 120% sulfation group. This may be due to a salting-out phenomenon with higher molecular weight polymer complexes. The data also demonstrated that KAFAK formed particles preferably with GAGs which are of a molecular weight and degree of sulfation similar to that of native GAGs.

Particles formed and were found to be stable within the 130–220 nm range. Particle diameter increased with both increasing degree of sulfation and molecular weight, indicating that both parameters may be utilized to adjust particle size using this method of particle formation. At molecular weights below 100 kDa, a predictable pattern formed where particles became larger as the degree of sulfation increased and as MW increased. This is shown in matrix of Figure 4, where green represents the lower end of particle size and red indicates larger particles. However, below an 80% degree of sulfation, particles did not form, with large, unstable aggregates formed instead (indicated by micrometer-scale diameter measurements).

**Figure 5:**
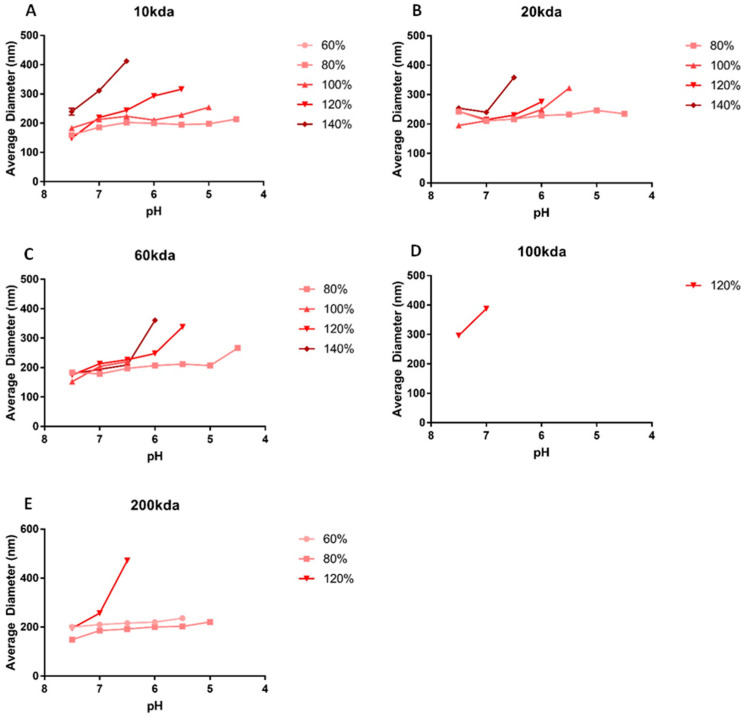
Average diameter of SHA-KAFAK nanoparticles at varying pH. Each graph represents a different MW to visualize the different behavior of different degrees of sulfation at the same MW. Data are grouped by the MW of SHA used (**A**–**E**), with each data set representing a different degree of sulfation.

**Figure 6:**
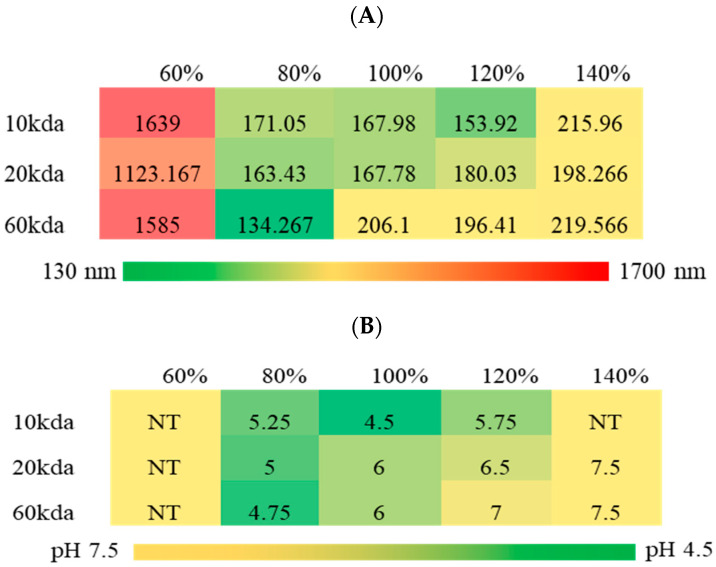
Matrix indicating (**A**) Average diameter and minimum stable pH of SHA-KAFAK-MK2i nanoparticles following 24 h incubation and addition of PBS pH 7.4. (**B**) Minimum stable pH of SHA-KAFAK nanoparticle groups below 100 kDa MW. ‘NT’ stands for Not Tested and represents particles that failed to meet minimum criteria for analysis.

To determine the stability of particles with respect to solution pH, the pH range from 7.5 to 4.5 was evaluated. The minimum stable pH was established as the pH at which the particles no longer had a unimodal distribution with a PDI < 0.2, indicating that they were separating from and releasing the peptide cargo. Particles were not tested below lysosomal pH of 4.5. Basic conditions were omitted due to a lack of physiological relevance. Particle size and zeta potential were measured at pH increments of 0.5. Distributions of particle size vs. pH can be seen in Figure 5. Particles made at 100% sulfation relative to that of DS showed pH sensitivity, with particles increasing in size as pH dropped. Particles created from SHA with a 120–140% degree of sulfation showed a rapid change in size with pH, indicating a strong sensitivity to pH with respect to particle diameter, and particles began separating and aggregating at relatively high pH. Conversely, SHA < 100% sulfation formed particles that were relatively unresponsive to pH and stable at quite low pH, indicating that higher degrees of sulfation may be used when cargo must be released at a higher pH and lower degrees of sulfation may be used when low pH release is necessary. The minimum stable pH and average particle diameter or each formulation are summarized in Figure 6. Minimum stable pH (i.e., release pH) showed that lower MW particles remained stable down to a lower pH and higher degree of sulfation, and high-MW particles exhibited less stability at low pH, aggregating before reaching a pH below 6. Figure 6B summarizes this finding, with green representing low pH (approaching 4.5) and yellow higher pH (near 7.5). Groups that were excluded from pH testing due to other stability issues have a minimum stable pH listed as 7.5 (unadjusted formulation pH) to facilitate visualization. Gradual increases in size over a range of pH values were likely due to swelling caused by changes in charge between the two molecules, either due to hydrolysis of the sulfates on the SHA, proximity to the charge isoelectric point, or charge shielding by the ions in solution.

In general, the behavior of larger SHA (>60 kDa) was unpredictable. Patterns in particle formation based on molecular weight are not as clear for particles formed from 100 or 200 kDa SHA as those formed from sHA in the range of 10–60 kDa. While this could be due to many factors, including polydispersity, it is plausible that generally, sHA with a molecular weight similar to that of native GAGs forms complexes with the KAFAK peptide that better support particle formation.

Taken together, these data show that the modulation of either or both the MW and degree of sulfation allows for control over particle size, with a range of 134 to 224 nm, as well as the minimum stable pH of the particles (below which point the particles will destabilize and the peptide will be released), across a range of 4.5 to 7. Modulating the degree of sulfation also gave control over swelling behavior, as indicated by the increasing slope at high degrees of sulfation in Figure 5; larger changes in particle diameter occurred with smaller changes in pH when the SHA was highly sulfated. This tunability matched or exceeded the size ranges and pH stability achievable with previously demonstrated systems [10,11,12,13,14,15,16,17]. Combined with our previous work, showing that particle sizes above 300–400 nm were achievable with electrospraying [28], this platform can deliver a myriad of particle sizes applicable to most use cases. This is key, as particle size has been shown to play in important role in systemic targeting, tissue permeation, and cellular uptake of nanoparticles and is, therefore, an important variable to control [37,38]. For example, nanoparticles with diameters greater than 200 nm are readily scavenged nonspecifically by monocytes and the reticuloendothelial system and may be ineffective [37]. Smaller particles, on the other hand, have been shown to permeate the vasculature of cancerous tissue, but not that of healthy tissue, allowing them to innately target cancerous regions in the body and deliver therapeutics [38]. The ability to control the pH at which the constituents separate is also useful for targeting as, for example, the pH of healthy, damaged, and cancerous tissue can vary [28,39,40], and this may be leveraged for targeted release. Tang et al. have shown that heparin-chitosan-PLGA nanoparticles can deliver heparin and fibroblast growth factor to damaged tissue specifically by taking advantage of this pH difference [10], although this system is limited not just by its complexity but also by the narrow pH response range (6.0 to 7.4) as well [10]. The encapsulated cargo may also be changed so long as it includes the KAFAK sequence, which has been shown to be the key driver of particle formation.

### 3.3. Enhancement of Biological Efficacy

The incorporation of peptides into particles can be critical to peptide stability when dosing in vivo. Peptides are intrinsic signaling molecules in the body and, as such, can serve as therapeutic interventions to treat dysfunctional biological processes. The delivery of therapeutic peptides is hampered by the numerous peptidases and excretory mechanisms that naturally attenuate hormonal signals in the body and drastically limit the half-life of peptides in the body. While essential for normal bodily function, this rapid degradation and clearance, along with extremely poor bioavailability when taken orally, limit the potential of therapeutic peptides. Complexation with GAGs into a nanoparticle using the method presented here has been shown previously to enhance peptide function, likely due to protection from the aforementioned degradative enzymes. To assess the biological activity of KAFAK-MK2i when complexed with SHA, five groups were selected for in vitro testing on inflamed keratinocytes.

Keratinocytes were chosen because they are key drivers of Atopic Dermatitis and respond strongly to the MK2i peptide, which inhibits the MK2 inflammatory pathway that is associated with the secretion of inflammatory cytokines and exacerbates inflammation [35]. Atopic Dermatitis is an inflammatory skin condition with an extremely high disease burden among children, affecting 20% of children globally, with no established, singular cause or cure [41,42], although it is speculated that dermal barrier dysfunction allows the ingress of debris and bacteria causing inflammation, itching, pain, flakiness, and skin damage. This chronic inflammation causes further barrier dysfunction, and the process continues [41,43,44,45,46,47,48]. One of these inflammatory pathways is the P38/MAPK pathway, which, when activated, upregulates the secretion of inflammatory cytokines such as IL-6 [2]. Delivery of the MK2i peptide has the potential to reduce the inflammatory response of keratinocytes by inhibiting MK2 (downstream of P38/MAPK) and to halt the progress of atopic dermatitis, if the peptide can be protected from enzymatic degradation, which is hastened in an inflammatory environment [49,50]. These nanoparticles should enhance MK2i’s performance compared to MK2i alone by protecting it from degradation. The different minimum stable pH and diameter of various particle groups may also confer higher efficacy by enhancing uptake and or lysosomal release of the peptide at low pH.

Hyaluronic Acid with MWs of 10 kDa, 20 kDa, and 60 kDa and degree of sulfation of 100% were chosen to determine the effects of MW on in vitro particle efficacy to evaluate a degree of sulfation similar to that of DS. Hyaluronic acid, MW 60 kDa, with 80, 100, and 120% degrees of sulfation were chosen to evaluate the effects of degree of sulfation, independent of MW, and MWs similar to that of DS. Differences between groups may suggest that charge ratio and molecular weight play a role in peptide efficacy when delivered from these particles. The groups evaluated here also represent nearly the entire range of particle sizes achieved.

**Figure 7:**
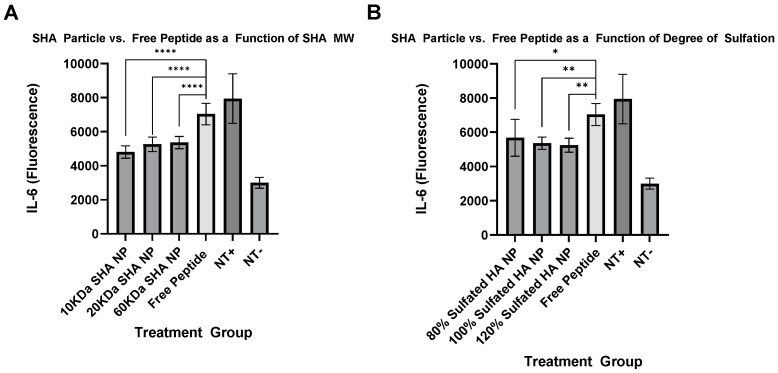
Five SHA-KAFAK-MK2i particle formulations were tested for their ability to suppress inflammation in keratinocytes as quantified by IL-6 secretion. Inflamed keratinocytes were stimulated with IL-1β and TGF-β (+) and subsequently treated with PBS (NT), SHA nanoparticles, or KAFAK-MK2i peptide alone in solution. As shown, loading into SHA particles improved MK2i efficacy and reduced IL-6 secretion at 24 h significantly below groups treated with unloaded KAFAK-MK2i peptide alone. The molecular weight of SHA and relative degree of sulfation of each particle formulation are listed. Data are grouped by (**A**) Three particle formulations of varying MW, but constant 100% degree of sulfation and (**B**) three particle formulations of constant, 60 kDa MW, but varying degrees of sulfation (****, **, * represent *p*-values less than or equal to 0.0001, 0.01, and 0.05, respectively).

Cells responded to all particle treatments similarly, as determined by IL-6 secretion, indicating that within the range tested, the function of KAFAK-MK2i was independent of pH of dissociation and particle size. Performance compared to the free peptide was significantly improved at 24 h after treatment in cells stimulated with IL-1β and TGF-β at 0.2 ng/mL and 10 ng/mL, respectively. Treatments are grouped as a function of SHA MW at a constant degree of sulfation (Figure 7A) and as a function of degree of sulfation at constant MW (Figure 7B).

While modulation of the pH stability and diameter did not result in significant changes in peptide performance, these data show that this nanoparticle platform provided a significant improvement in peptide efficacy. We hypothesize that this in vitro model was not sensitive to these two variables and the difference in performance of the free peptide and nanoparticles was due to protection of the cargo, expanding its half-life in vitro in the presence of serum, thus allowing intact peptide to enter cells. This is supported by the fact that, in the absence of fetal calf serum, both free peptide and nanoparticles exhibited excellent performance and strongly suppressed inflammation but, in the presence of serum, NPs significantly outperformed the free peptide [2].

**Figure 8:**
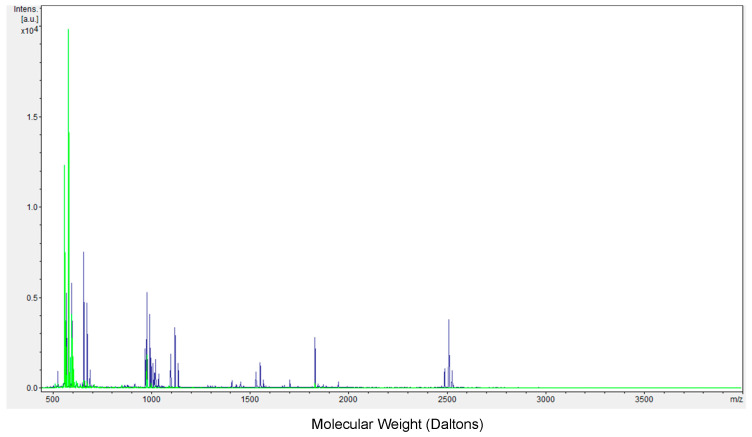
Mass distribution of peptide fragments obtained using MALDI-TOF following enzymatic assault on KAFAK-MK2i alone (green) and SHA + KAFAK-MK2i nanoparticles (blue).

To directly test if these nanoparticles did in fact protect MK2i from degradation by enzymes, peptide alone and SHA + KAFAK-MK2i particles were challenged with enzymatic degradation using TrypLE, a recombinant trypsin substitute. KAFAK-MK2i and SHA + KAFAK-MK2i nanoparticles were incubated with 10% TrypLE for 10 min with agitation at 37 °C. Peptide concentration was the same in both groups. The formation of peptide fragments was assessed using MALDI-TOF. As shown in Figure 8, KAFAK-MK2i alone was entirely lysed into small fragments in the presence of TrypLE with no peaks appearing at 2487 (green trace), the intact MW of KAFAK-MK2i. Nanoparticles, on the other hand, showed some remaining whole peptide, as well as several fragments with MW between 1000 and 2487 Da (blue trace), indicating that the particles provide some resistance to proteolysis even at high enzyme concentration, and supporting the hypothesis that electrostatic complexation with GAGs conferred some protection from enzymatic degradation.

The binding properties of sulfated glycosaminoglycans have been exploited previously, mostly commonly to load and control release of growth factors, in bulk hydrogel and in particle formulations. The incorporation of sulfated hyaluronic acid to collagen hydrogels, for example, has been shown to mediate the binding of Epidermal Growth Factor and slow its release, thereby enhancing wound healing by inducing keratinocyte migration and Epidermal Growth Factor receptor signaling [51]. Similarly, highly sulfated chitosan nanoparticles were found to load heparin binding epidermal growth factor and promote its release while maintaining its structural integrity, leading to complete wound closure in a mouse model. Sulfated hyaluronic acid was also found to bind with high affinity to Bone Morphogenic Protein 4, while sulfated cellulose bound and released Bone Morphogenic Protein 2 and Basic Fibroblast Growth Factor and improved their cellular activities [52,53,54]. These systems, however, use the natural affinity of sulfated GAGs and growth factors and offer no ability to change the cargo and do not demonstrate protection from lysis or an ability to modify loading and release behavior. The sulfated-HA + KAFAK nanoparticle system described here has the potential to load a myriad of cargo and protect the cargo from enzymatic degradation, while remaining fast, and cheap, and offering the tunability of particle size and dissociative properties. GAG-based therapeutic molecules have also shown promise in recent years for the treatment of inflammatory conditions such as vascular reperfusion injury and post-traumatic osteoarthritis [55,56,57]. Looking forward, this technology may be expanded to incorporate therapeutic peptides or other cargo into particles with GAG-based therapeutics to make dual-action nanoparticles capable of suppressing inflammation via two distinct mechanisms. More exploration is needed with more cargo types and differing GAGs to fully understand the opportunities this technology offers.

## 4. Conclusions

Here, we present a nanoparticle platform which, through the inclusion of the KAFAK amino acid sequence, allows peptides, and potentially other cargo, to be complexed into a nanoparticle with glycosaminoglycans quickly and easily, with control over diameter and pH stability. Complexation into a nanoparticle using this method confers protection from degradation and, thereby, improves the efficacy of the encapsulated peptide. By modulating the degree of sulfation of hyaluronic acid, a naturally unsulfated GAG, the pH at which the peptide cargo is released can be modulated between 4.5 and 7 and particle diameter can be modulated between 134 nm and 220 nm. These nanoparticles were tested in vitro in inflamed keratinocytes to assess their ability to suppress cytokine secretion by resident keratinocytes in atopic dermatitis. SHA + KAFAK-MK2i particles significantly outperformed free peptide in suppressing the expression of IL-6, a marker of inflammation, after 24 h of treatment following a 1-h inflammatory assault with IL-1β and TGF-β thanks to protection from enzymatic degradation. The conferral of protection via complexation with GAGs was also confirmed via MALDI-TOF following enzymatic assault.

This platform has the potential to accelerate applications of GAG-based nanoparticles as drug delivery and release systems through the simplification of the particle formation process, while retaining the control over particle properties not possible with other systems. It also abrogates the need for synthetic polymers used in other electrostatic nanoparticle systems [10,11,12,13,14,15,16,17]. More work is needed to show whether this can be applied to larger molecules, such as growth factors, and to elucidate the limitations of cargo that may be encapsulated. Preliminary experiments show that fluorescently labeling KAFAK-MK2i with fluorescein does not affect particle formation. Fluorescein, an anionic molecule, increases the peptide MW by 13.4%, and is shown to affect protein-polysaccharide binding [36] and affect peptide performance in vitro [58]. These data indicated that this particle formulation method was not affected by the modification of the cargo and could be used for a myriad of peptides. It also suggested that the encapsulation of small molecules, conjugated with KAFAK, may be possible. While this would add the complexity of synthesizing a drug–peptide conjugate, small molecule–GAG conjugates are already in use in more complex nanoparticle systems, using amino acids as cleavable linkages [17]. At the very least, this technique can be applied to the protection of a myriad of therapeutic peptides by simply including KAFAK in the amino acid sequence, while providing control over particle properties, which are essential for targeting and targeted release.

## Figures and Tables

**Table 1:** Summary of peptide sequences, function, and particle-forming ability. Colored letters indicate charged amino acids.

Binding Sequence	Cargo (MK2i)	Cargo MW (Da)	Stable Particle Formation?
KAFAKLAARLYR	KALARQLGVAA	2487.02	Yes
KAFAKLAARLYR	KALARQLGVAA-FITC	2819.33	Yes
YARAAARQARA	KALARQLGVAA	2283.65	Yes

## Data Availability

All raw data presented herein are available upon request.
